# Detection of natural *Trichinella murrelli* and *Trichinella spiralis* infections in horses by routine post-slaughter food safety testing

**DOI:** 10.1016/j.fawpar.2018.06.001

**Published:** 2018-06-02

**Authors:** Brad Scandrett, Kelly Konecsni, Laura Lalonde, Pascal Boireau, Isabelle Vallée

**Affiliations:** a Centre for Food-borne and Animal Parasitology, Canadian Food Inspection Agency, Saskatoon Laboratory, 116 Veterinary Road, Saskatoon, Saskatchewan S7N 2R3, Canada; b UMR BIPAR, ANSES, Ecole Nationale Vétérinaire d'Alfort, INRA, Université Paris-Est, Animal Health Laboratory, 14 rue Pierre et Marie Curie, F-94 701 Maisons-Alfort, France

## Abstract

*Trichinella spiralis* typically infects domestic swine, wild boar and occasionally horses, has a cosmopolitan distribution, and consequently is most frequently associated with food-borne outbreaks of trichinellosis in humans. *Trichinella murrelli* is typically found in wild carnivores in temperate areas of North America, where it has been responsible for outbreaks of human trichinellosis due to consumption of infected wild game. There has previously been only indirect evidence of natural infection with *T. murrelli* in a horse originating from Connecticut and implicated in an outbreak of trichinellosis in France in 1985. We describe a *T. murrelli* infection detected during routine testing of a horse from the USA imported to Canada for slaughter and export to the European Union (EU). Approximately 5 or more larvae per gram were recovered from digested tongue and diaphragm samples and identified as *T. murrelli* by PCR. This case provides the first direct evidence of naturally acquired *T. murrelli* infection in a horse, and further supports the potential food safety risk posed by this parasite species. It is the first instance in Canada of the detection of a *Trichinella*-infected horse via routine post-mortem testing. *Trichinella spiralis*-infected horses have been similarly detected by regulatory testing in France, and further details of two such previously reported cases are also provided here. The cases described herein underscore the importance of continued vigilance in quality assured food safety testing of horse meat to mitigate the risk of human trichinellosis.

## Highlights

• First direct evidence of natural infection with *Trichinella murrelli* in a horse
• First detection in Canada of a *Trichinella*-infected horse via regulated testing
• *Trichinella murrelli* should be considered a potential food safety risk in horsemeat.
• Horse tongue and diaphragm are effective for detecting *T. murrelli* and *T. spiralis*.
• Screening of horse carcasses by digestion assay mitigates risk of trichinellosis.

## 1 Introduction

Zoonotic nematodes of the genus *Trichinella* infect a variety of mammalian, avian or reptilian hosts and have a direct life cycle with transmission to new hosts occurring via the consumption of meat containing infective first stage larvae. *Trichinella spiralis*, one of 12 taxa currently recognized worldwide, is the etiological agent of the domestic cycle maintained primarily in pigs and consequently is responsible globally for most outbreaks of human trichinellosis, acquired from infected pork (Murrell and Pozio, 2011). *Trichinella murrelli* is prevalent in mammalian wildlife in temperate areas of North America, primarily the USA, where it has been responsible for outbreaks of human trichinellosis due to the consumption of infected wild game (Hall et al., 2012; Pozio and La Rosa, 2000). Only one occurrence of *T. murrelli* has been reported in Canada, in a wild cougar (*Puma concolor cougar*) from British Columbia (Gajadhar and Forbes, 2010), although the parasite has also been recovered from a red fox (*Vulpes vulpes*) and raccoon (*Procyon lotor*) from southern Ontario, Canada (B. Scandrett, unpublished data) and is likely present in a variety of Canadian wildlife species, particularly in those regions closest to the border with the USA.

Since 1975, horse meat has been implicated in a number of European outbreaks of human trichinellosis, and there are numerous reports of natural infections of horses with *T. spiralis*, and to a lesser extent with the sylvatic species *T. britovi* (Arriaga et al., 1995; Boireau et al., 2000; Liciardi et al., 2009). From a 1985 outbreak of human trichinellosis in France there was also indirect evidence suggesting the source to be a horse from Connecticut, USA naturally infected with *T. murrelli*, but this is apparently the only such report of *T. murrelli* in a livestock host species (Ancelle et al., 1988; Dupouy-Camet et al., 1994; Pozio, 2015). To our knowledge, this current paper therefore describes the first confirmed case of naturally acquired *T. murrelli* infection in a horse, or in any livestock host, and is the first report of the detection of *Trichinella*-infected horse meat in Canada during routine post-slaughter testing for export. Further details for two previously reported cases of *T. spiralis*-infected horses similarly detected by routine post-slaughter testing in France will also be provided herein (Boireau et al., 2001).

## 2 Materials and methods

### 2.1 Epidemiological information

On September 12, 2012, one horse (a six-year old mare) from a shipment of 34 animals imported from the USA for immediate slaughter at a federally-registered establishment in Alberta, Canada was found to be infected with *Trichinella* larvae during routine post-slaughter testing. Testing was performed by an on-site laboratory certified by the Canadian Food Inspection Agency (CFIA) in accordance with requirements for export of horse meat to the European Union (EU) (European Union, 2015; Forbes et al., 2005). Additional samples were collected for the National Reference Laboratory (NRL; CFIA Saskatoon Laboratory, Centre for Food-borne and Animal Parasitology) for confirmatory testing and determination of larval loads in various carcass sites; the infected carcass was subsequently condemned and decontaminated via rendering. Results of *Trichinella* testing for all other carcasses in the shipment were negative. The United States Department of Agriculture (USDA) was notified of these findings, and provided with information on the infected animal obtained from the United States Origin Health Certificate which accompanied the imported shipment to facilitate any further investigation as warranted.

Similarly, in October 1999 and March 2001, two horses imported to France from Poland and Serbia, respectively, were each found to be infected with *Trichinella* during routine testing by two independent qualified laboratories in France (Boireau et al., 2001). The horse from Poland was a seven-year old mare from a farm close to the Russian border, and sent to a slaughterhouse in Carpentras (Vaucluse, France). The second horse (age and gender unknown) was purchased from a horse-market in the city of Ruma (Srem district, Serbia) and sent to a slaughterhouse in Pézenas (Hérault, France). Both carcasses were removed from the food chain and samples also obtained by the French NRL (Anses, Animal Health Laboratory, Maisons-Alfort) for confirmatory testing and assessment of parasite burden in various carcass sites.

### 2.2 Diagnostic sample collection and digestion assay

Samples collected from the imported horses slaughtered in Canada for routine testing by the certified laboratory for export qualification were taken from the caudoventral tongue muscle, which is a predilection site for *Trichinella* infection in the horse (Gamble et al., 2000). Samples of a minimum of 5 g each were pooled for *Trichinella* testing by the artificial digestion method as recommended by the World Organisation for Animal Health (OIE) and International Commission on Trichinellosis (ICT), using a previously validated double separatory funnel method (Forbes et al., 2008; ICT, 2011; OIE, 2017). The presence/absence of larvae, as well as the number of any larvae detected and their appearance (motile/non-motile) was recorded for each digest. If nematodes morphologically consistent with *Trichinella* first stage larvae (L1) were detected in the initial sample pool, successive digests of decreasing numbers of pooled samples of 10 g tongue or 20 g of diaphragm crus (an alternate predilection site) were performed to deduce the infected carcass(es). Confirmatory digests by the NRL in Saskatoon were performed on 10 g tongue and 20 g diaphragm samples from each suspect carcass, and trichinelloscopy also performed on representative samples of tongue and diaphragm.

The initial testing of the horses from Poland and Serbia slaughtered in France entailed similar pooled digestion of 10 g of apex tongue muscle and 10 g of diaphragm per carcass by each of two independent certified laboratories (in Nimes and Avignon for the Polish horse; in Marseille and Montpellier for the Serbian horse) in accordance with EU and French legislations at that time (European Communities, 1976 and Ministère de l'agriculture, de l'alimentation, de la pêche et des affaires rurales, 1998, respectively). For suspect-positive pools, individual digests of at least 20 g of tongue or diaphragm were performed for each carcass contributing to the pool. Confirmatory analysis was carried out at the French NRL by artificial digestion of 50 g of tongue from each suspect carcass. Histopathological examination was also performed on tongue muscle samples from the Serbian horse, following a standard technique for fixation and imbedding in paraffin.

### 2.3 Molecular identification

Representative larvae from the positive horse detected in Canada were washed with PBS and stored in PCR buffer (10 mM Tris-HCl, pH 8.3, 50 mM KCl, 1.5 mM MgCl_2_) until extraction later the same day; all remaining larvae were similarly processed and stored at −70 °C in PCR buffer. The identity of recovered larvae from the *Trichinella*-positive horses in Canada and France was determined by the respective NRLs using multiplex PCR as previously described (Gajadhar and Forbes, 2010; Zarlenga et al., 1999). Modifications implemented for the larvae recovered in Canada were as follows: DNA extraction from L1 s was performed in 15 μL of PCR buffer heated to 90 °C for 10 min and then placed on ice for 5 min. Samples were then incubated for 6 h with 2 μL proteinase K (20 mg/mL; Qiagen, Toronto, ON). Following incubation, the samples were heated to 90 °C for 10 min, then centrifuged at 10,000 x g for 5 min. Recovered DNA was then subjected to PCR or stored at −20 °C until tested. The PCR reaction mixture contained 1× Amplitaq Gold 360 master mix (Applied Biosystems, Foster City, CA), 50 uM total concentration primer pairs 1–5, 2 μL GC enhancer (Applied Biosystems, Foster City, CA), 2.5 μL genomic DNA and adjusted with sterile dH20 to a final volume of 25 μL. PCR cycling conditions using a BioRad C1000 thermal cycler (BioRad, Hercules, CA) were as follows: 10 min at 95 °C, followed by 40 cycles of denaturation at 95 °C for 15 s, annealing at 55 °C for 30 s, extension at 72 °C for 90 s, then a final extension step at 72 °C for 7 min. PCR products were separated on a 2.5% high resolution agarose gel and detected using SYBR Gold (Invitrogen, Carlsbad, CA). Recovered larvae from the Polish and Serbian horses were submitted to the International *Trichinella* Reference Centre (Istituto Superiore di Sanità, Rome, Italy) for inclusion in their database of *Trichinella* reference strains.

### 2.4 Distribution of larvae in various carcass sites

To assess the baseline *T. murrelli* larval burdens in various carcass sites of the infected horse slaughtered in Canada, additional tongue and diaphragm tissues were collected, as well as neck, trapezius, supraspinatus, triceps brachii and rectus abdominis muscle. Digests of 100 g were performed in triplicate at the NRL for each tissue site, except tongue where a total of only approximately 27 g tongue tissue was available for this purpose. For the *T. spiralis*-infected horses slaughtered in France, the same muscle sites as above, except for rectus abdominis and neck, were examined, in addition to the obliquus externus abdominis. Masseter muscle was also examined from the horse sourced from Poland. Individual digests were performed on 100 g from each muscle site for the Polish horse and 50 g for the Serbian horse (except for trapezius for which only approximately 29 g was tested). In all cases, the number of larvae per gram (lpg) was determined for each site by dividing the total number of larvae recovered by the total weight of sample tested, including any samples tested by the respective NRL to confirm the original diagnosis.

### 2.5 Bioassay

To assess the viability and infectivity of the recovered *T. murrelli* larvae, mouse and rat bioassays were performed at the Canadian NRL using larvae obtained from digested diaphragm or neck muscle. Seven female CD-1 mice were orally administered approximately 50 (1 mouse), 100 (2 mice), or 500 (4 mice) motile or tightly coiled (i.e. presumably viable) larvae and two female Sprague-Dawley rats each similarly received approximately 250 larvae. All mice and rats were killed within 2 years post-inoculation and tissues digested and examined for *Trichinella* larvae.

The recovered *T. spiralis* isolates from one or more of the available muscle sites were similarly bioassayed at the French NRL by oral inoculation of mice. Two female OF1 mice were inoculated per isolate, with 300 or 120 motile/tightly coiled larvae from the Polish or Serbian horse, respectively. Mice were killed 3 months post-inoculation, and muscle tissues digested and examined for *Trichinella* larvae.

## 3 Results

Larvae morphologically consistent with *Trichinella* were detected during routine testing of tongue and diaphragm from one of the carcasses of the horses imported into Canada, with average infection intensities of approximately 5 and 8 lpg, respectively. The initial individual digests of submitted tongue and diaphragm performed by the NRL to confirm the diagnosis and source carcass yielded an average of approximately 5 lpg in both sample types. Most of the recovered larvae appeared motile or tightly coiled, indicating viability. All larvae examined via compressorium were encapsulated, and PCR identified the species as *Trichinella murrelli*. Similar individual digests of tongue and diaphragm from the Polish and Serbian horses yielded overall averages of 6.2 and 2.5 lpg, and of 4.8 and 0.7 lpg, respectively. Genotyping performed on these two isolates identified them as *T. spiralis* (ISS codes 889 and 1100 for the isolates from the Polish and Serbian horse, respectively). Histological examination of tissue sections containing the Serbian isolate demonstrated *Trichinella* larvae within a nurse cell exhibiting a thick collagen capsule. The overall *T. murrelli* and *T. spiralis* larval burdens determined for all assessed muscle sites in the respective carcasses are indicated in Table 1.

**Table 1:** Parasite burden in select muscle sites for 3 horses naturally infected with *Trichinella spiralis* or *T. murrelli*, as determined by digestion assay. Horses A, B and C originated from the USA, Serbia and Poland, respectively.

Muscle site	Number of *Trichinella* first stage larvae (L1) per gram		
	Horse A *T. murrelli*	Horse B *T. spiralis*	Horse C *T. spiralis*
Tongue	4.6	4.8	6.2
Masseter	NA^a^	NA	0.5
Diaphragm	6.8	0.7	2.5
Triceps brachii	1.1	0.6	0.3
Supraspinatus	1.0	0.2	0.3
Rectus abdominus	0.8	NA	NA
Obliquus externus abdominis	NA	0	0.03
Trapezius	0.5	0.2	0
Neck	0.5	NA	NA

a Tissue not available.

All *T. murrelli* mouse bioassays yielded live larvae on digestion assay. The four mice inoculated with 500 L1 each were killed 489–538 days post-inoculation (dpi) and had infection intensities of 9.7, 28.8, 34.4, and 95.5 lpg. The two mice inoculated with 100 L1 each were killed at 94 and 471 dpi and had infection intensities of 4.3 and 0.8 lpg, respectively. The single mouse inoculated with 50 L1 was killed at 409 dpi and yielded 5.0 lpg. Neither of the two rats, killed at 587 and 616 dpi, yielded any larvae on digestion assay. All *T. spiralis* mouse bioassays similarly yielded live larvae. The two mice inoculated with larvae from the Polish horse enabled the collection of 47,000 L1 (ISS 889), whereas 15,000 L1 (ISS 1100) were obtained from mice infected with the Serbian isolate.

## 4 Discussion

Natural infection with *Trichinella* in horses is uncommon, which is not surprising given that horses are herbivores and this parasite requires the consumption of infected meat for transmission to a new host. However, natural infections do occur, with most reported cases due to *Trichinella spiralis* (Pozio, 2015). In the EU, from 1975 to 2001 it has been estimated that less than one in 250,000 horses were infected with *Trichinella*; from 2002 onwards this estimate has dropped to approximately one in 750,000, presumably as a result of the overall reduction in *Trichinella* infection in domestic pigs in Eastern Europe (Pozio, 2015). In most instances, these infections are probably acquired through inadvertent ingestion of infected meat; however, intentional feeding of meat to horses by owners for conditioning purposes has also been described (Murrell et al., 2004). The epidemiological investigations conducted for the infected Polish and Serbian horses presented in this study did not indicate the latter, so accidental ingestion of infected rodent tissue with the livestock feed provided was a more likely scenario. The import health certification for the *T. murrelli*-infected horse in the current report attested to its origin in the USA. Although no further details were available regarding the origin and history of this animal in the USA, it is probable that this horse was infected with *T. murrelli* via ingestion of infected tissue from a wildlife host species in an endemic region. Between 2007 and 2016, approximately 80,000 horses were being slaughtered annually in Canada, with a substantial proportion imported from the USA (Agriculture and Agri-Food Canada, 2018; Whiting, 2007).

Although several studies of experimental *T. spiralis* infection in horses have been undertaken, only one (Soulé et al., 1989a, Soulé et al., 1989b) has been conducted of *T. murrelli*, and used a human biopsy-sourced isolate from the aforementioned 1985 outbreak in France linked to a horse from the USA (Boireau et al., 2000). That study referred to the isolate used (CTRD 85 strain) as *T. spiralis nativa*, but it was subsequently demonstrated to be *T. murrelli* (Dick et al., 1990; Pozio and La Rosa, 2000; Pozio et al., 2001). The study found that horses were less susceptible to *T. murrelli* and infective larvae persisted in the tissues for a shorter duration compared to *T. spiralis*. Since the majority of *T. murrelli* larvae recovered in the current case were alive, this suggests that the infection was relatively recent. The positive mouse and negative rat bioassay results for *T. murrelli* in this study are consistent with the lower reproductive capacity indices reported for rats (0.7–2.4) compared to mice (1.2–9.5) experimentally inoculated with this species (Pozio and La Rosa, 2000). However, these bioassay results must be interpreted with caution due to the relatively low numbers of larvae administered and the protracted post-inoculation periods after which the larval loads were assessed.

For food safety purposes, the reliable detection of carcasses harbouring ≥1 lpg *Trichinella* spp. is recommended (OIE, 2017). Thus the *T. murrelli* larval burdens in this case could have posed a food safety risk to consumers, particularly in the EU to which the infected carcass was destined, where consumption of raw or undercooked horse meat is customary in France and Italy (Boireau et al., 2000). Similarly, although the histological finding of larvae encapsulated within nurse cells with thick collagen walls indicates an infection of some chronicity for the Serbian horse, the recoveries of infective larvae and infection intensities for both *T. spiralis* cases described here suggest these could also have constituted a public health risk.

Of the five common carcass sites assessed in all 3 infected horses, the diaphragm and tongue yielded the highest *T. murrelli* or *T. spiralis* burdens. This is consistent with the results of the experimental infections with *T. murrelli* conducted by Soulé et al., 1989a, Soulé et al., 1989b, and with those for natural and experimental infections with *T. spiralis* (Gamble et al., 1996), and supports the continued testing of these predilection sites for the detection of *Trichinella* spp. in horse meat. Although masseter is also a recommended predilection site, this was only available for assessment from the *T. spiralis*-infected horse from Poland. However, the relatively low recovery of 0.49 lpg from the masseter in this carcass, compared to the much higher lpg values for tongue and diaphragm, highlights the biological variation that can be encountered for individual animals, even amongst recognized predilection sites (Kapel et al., 2005; Pozio et al., 1999). Collecting samples primarily near tendinous junctions may have increased the numbers of larvae recovered (European Union, 2015), but was not done consistently for the various sites assessed in this study.

Although the prevalence of *Trichinella* in horse meat remains low, a single infected carcass can result in an outbreak of trichinellosis affecting hundreds of people, with *T. spiralis* and *T. britovi* having posed the greatest risk in this regard (Boireau et al., 2000; Pozio, 2015). The current report provides the first direct evidence of naturally acquired *T. murrelli* infection in a horse, and thus further supports the potential for this species to also pose a food safety risk in horse meat. This is also apparently the first instance of the detection of a *Trichinella*-infected horse during post-slaughter screening in Canada and exemplifies the importance of quality assured food safety testing in mitigating the risk of trichinellosis to consumers.

## Conflict of interest

The authors have no conflict of interest.
